# Inhibition of Iron Release from Donkey Spleen Ferritin through Malt-Derived Protein Z–Ferulic Acid Interactions

**DOI:** 10.3390/foods12020234

**Published:** 2023-01-04

**Authors:** Mingyang Sun, Hanhan Liu, Chen Xu, Zhenghui Jiang, Chenyan Lv

**Affiliations:** College of Food Science & Nutritional Engineering, China Agricultural University, Beijing Key Laboratory of Functional Food from Plant Resources, Beijing 100083, China

**Keywords:** ferritin, protein Z, interaction, ferulic acid, iron release

## Abstract

Protein–small molecule interactions naturally occur in foodstuffs, which could improve the properties of protein and small molecules. Meanwhile, they might affect the bioavailability and nutritional value of proteins. Ferritin, as an iron-storage protein, has been a focus of research. However, the complexity of foodstuffs enables the interaction between ferritin and food components, especially polyphenols, which can induce iron release from ferritin. Thus, the application of ferritin in food is limited. Inspired by the natural-occurring, strong protein–polyphenol interactions in beer, to inhibit the iron release of ferritin, the malt-derived protein Z (PZ) was chosen to interact with ferulic acid (FA), an abundant reductant in malt, beer, and other foodstuffs. The analysis of the interaction between PZ and FA was carried out using fluorescence spectroscopy, the results of which suggest that one PZ molecule can bind with 22.11 ± 2.13 of FA, and the binding constant is (4.99 ± 2.13) × 10^5^ M^−1^. In a molecular dynamics (MD) simulation, FA was found to be embedded in the internal hydrophobic pocket of PZ, where it formed hydrogen bonds with Val-389 and Tyr-234. As expected, compared to iron release induced by FA, the iron release from donkey spleen ferritin (DSF) induced by FA decreased by 86.20% in the presence of PZ. Meanwhile, based on the PZ–FA interaction, adding PZ in beer reduced iron release from DSF by 40.5% when DSF:PZ was 1:40 (molar ratio). This work will provide a novel method of inhibiting iron release from ferritin.

## 1. Introduction

Protein–small molecule interactions in nature are a universal phenomenon. For example, heme interacts with proteins in mammalian cells as part of the prosthetic group to perform its physiological functions [1]. In addition, human serum albumin could combine fatty acids for storage and transportation [2]. Interestingly, protein–small molecule interactions occur commonly in foodstuffs as well, particularly protein–polyphenol interactions. Such interactions could improve protein properties such as thermal stability, surface hydrophobicity, and structural stabilization, while protecting small molecules against light-, thermal-, and pH-induced damage [3,4,5].

However, protein–small molecules can decrease the bioavailability and nutritional value of protein as well. Ferritin is a kind of iron storage protein, which could accommodate up to ~4500 iron atoms in the form of the iron core stored in the cavity. Natural components in foodstuffs, especially phytochemicals, could induce iron release from ferritins, such as FMNH2, FADH2, ascorbate, epigallocatechin gallate, and other phenolic acids [6,7,8,9,10,11,12]. The released iron could cause quality deterioration of food such as iron smell and discoloration. In addition, it is reported that under the reaction of ferritin with reductants, the cleavage and mutation of DNA increased via a Fenton reaction of free iron ions released from ferritin [13]. To prevent iron release, we chose beer as a reducing agent and fabricated a donkey spleen ferritin–pectin complex with electrostatic interaction in previous research, and the iron release from ferritin in beer was significantly reduced by 52.68% [14]. However, exogenous pectin additives are unstable, which might increase the viscosity and affect the taste of food. Therefore, selecting natural-occurring components that can form a stable complex with phytochemicals and reduce iron release is of vital importance. Ferulic acid (FA), as a major hydroxycinnamic acid, is widely distributed in plant foods, especially in coffee, wheat bran, barley malt, and beer [15,16,17,18]. FA is the most abundant phenolic acid in beer, the contents of which range from 83.9 ± 0.35 to 296.02 ± 4.88 μg/100 mL [18,19]. Meanwhile, ferulic acid has been proven to induce ferritin to release iron [14]. Of all the interactions in beer components, the protein–polyphenol interaction contributes an important role in beer’s stability, flavor, and visual appearance. Inspired by the protein–polyphenol interactions occurring in beer, it is possible to find a kind of protein that can bind with ferulic acid naturally.

Proteinaceous materials in beer primarily originates from malt, and proteins are essential for the quality of beer [20]. Among them, ~9 kDa lipid transfer protein (LTP) and ~43 kDa protein Z (PZ) stay soluble and have shown good stability in resisting proteolysis and heating [21]. Former research has demonstrated that LTP1 interacts with polyphenols via hydrophobic interactions and hydrogen bonds. The high level of proline residues in LTP1 makes it possible to form hydrogen bonds with hydroxyl groups in polyphenols due to the exposed carbonyl oxygens in the protein backbone [22].

The primary protein in beer is PZ, which comes from barley malt. In addition to its role in maintaining foam stability, it functions in physiological metabolism such as inflammation, coagulation, thrombosis, and immunity. Recently, researchers have concentrated on PZ as a promising carrier; so far, PZ as a carrier has enhanced the solubility, stability, and bioavailability of xanthohumol, curcumin, and other bioactive compounds [23,24,25]. Based on previous research, we supposed that the naturally occurring protein PZ in beer might interact with ferulic acid to reduce iron release from ferritin. However, the interaction between ferulic acid and PZ is lacking.

In this study, we extracted and purified the PZ derived from barley malt and explored the interaction between PZ and FA using fluorescence spectroscopy, synchronous fluorescence spectroscopy, circular dichroism (CD) spectra, and fluorescence resonance energy transfer (FRET). In order to understand how specifically FA interacts with PZ, molecular dynamics (MD) was used to analyze potential binding sites and forces. Finally, iron release from the donkey spleen was evaluated in the presence of PZ. It is hoped that this work may shed light on the interaction between food components and the prevention of iron release from ferritin.

## 2. Materials and Methods

### 2.1. Samples and Chemicals

Donkey spleens, barley malt, and Qingdao beer were purchased from a local market in Beijing, China. Ferulic acid (>99.0% purity) and ferrozine were obtained from Solarbio (Beijing, China), while Macklin (Shanghai, China) supplied ammonium sulphate (SA). The markers for SDS-PAGE and native PAGE were obtained from Biodee (Beijing, China). DEAE Sepharose Fast Flow was obtained from GE Healthcare Bio-Sciences AB (Beijing, China). In this work, ultrapure water was used along with other analytical-grade chemicals and solvents.

### 2.2. Preparation of Protein Z and Donkey Spleen Ferritin

Protein Z (PZ) was purified as previously reported [26]. The barley malt was crushed, and proper ultrapure water was added to obtain a wort. After filtration, the supernatant was precipitated with a gradient of 40–60% SA and then stored at 4 °C overnight. In addition, protein precipitates were obtained via centrifuging at 8000 rpm for 20 min, followed by dissolution in 50 mM Tris-HCl (pH = 7.5). To remove the other proteins, it was heated at 90 °C for 30 min, then centrifuged at 10000 rpm for 10 min. After dialyzing in 50 mM Tris-HCl (pH = 7.5), DEAE Sepharose Fast Flow and Sephacryl S-300 were used to purify crude PZ. Donkey spleen ferritin (DSF) was purified as referred to in previous methods [14]. The fresh donkey spleen was pretreated and cut into pieces. Subsequently, the spleen was homogenized well with ultrapure water (1:3, *m/v*). After filtering, the solution was heated to 80 °C for 10 min, centrifuged at 8000 rpm for 20 min, and the precipitation dissolved in Tris-HCl (pH 7.5, 50 mM). Next, it was eluted from a DEAE-Sepharose Fast Flow column with a NaCl gradient from 0 to 1.0 M.

Purification was identified using SDS-PAGE and native PAGE, and the concentration protein was measured using a Lowry protein assay kit.

### 2.3. Fluorescence Spectroscopic Analysis

The fluorescent spectrums were recorded with a spectrophotometer (Cary, Varian, Palo Alto, PAMF, USA). The 1.0 μM of PZ was prepared in a sodium citrate buffer (50.0 mM pH 4.2). During the titrations, 0.5 μL of FA (10 mM) was added continuously to the 1.0 mL PZ solution. Samples were excited at 280 nm, and the emission spectra were recorded from 290 to 600 nm with 10 nm widths for the excitation and emission slits. The synchronous fluorescence spectra of solutions were scanned from 200 to 400 nm after setting the Δλ between the excitation and emission wavelengths at 15 or 60 nm. The excitation and emission slit widths were set at 10.0 and 5.0 nm, respectively. The concentration of PZ was 4.0 μM in the sodium citrate buffer (50.0 mM, pH 4.2). Titrations were performed by continuously adding 2 μL of FA (10 mM) to a PZ solution (1 mL) with concentrations ranging from 20 to 120 mM.

### 2.4. Circular Dichroism Spectra Measurement

Circular dichroism (CD) spectra of PZ (0.1 mg/mL in PB buffer, pH 4.2) and PZ–FA (molar ratio of PZ and FA from 1:5 to 1:30, PB buffer, pH 4.2) were recorded on a Chirascan spectrometer (Applied Photophysics Inc., Leatherhead, Surrey, UK) in the far-UV region (190–260 nm) at 25 °C. The samples were measured in a quartz cell with a path length of 1 mm, and each sample was scanned with 3 replications. The analysis of CD spectra data was performed using CDNN and Chirascan software.

### 2.5. Molecular Dynamics (MD) Simulation

The TR-Rosetta website (https://yanglab.nankai.edu.cn/trRosetta, accessed on 1 October 2021) was applied to predict the structure of PZ since it lacks crustal structure, which is based on its amino acid sequence (resource: *Hordeum vulgare*) obtained from NCBI (https://www.ncbi.nlm.nih.gov/protein/, accessed on 1 October 2021). The ferulic acid conformer was obtained from the Pub Chem databank (https://pubchem.ncbi.nlm.nih.gov, accessed on 1 October 2021). The receptor (PZ) and the ligand (FA) were pretreated, and then molecular docking was performed using a Schrodinger Maestro v11.5 to obtain conformation of the PZ–FA complex [27].

Molecular dynamics (MD) simulation was explored, as previously reported [25]. MD analysis of the PZ–FA interaction was performed using the AMBER 18, based on ff14sb and GAFF2 force fields, respectively [28,29,30]. After that, hydrogen atoms were added to the simulated system, which was put into a truncated octahedral TIP3P solvent box with an edge length 10 Å from the edge of the simulation box. Following this, Na^+^ and Cl^+^ ions sufficient for system charge equilibrium were added. To minimize the system energy, the steepest descent algorithm and the conjugate gradient algorithm, both for 2500 steps, were used before MD simulations. After the minimization step, heating simulation was performed by linearly increasing the temperature (200 ps) from 0 K to 298.15 K at a constant volume. Then, the system was equilibrated by keeping the system for 500 ps NVT simulation at 298.15 K and 500 ps NPT simulation. Then, MD simulations were conducted at NPT for each system for 100 ns. The truncation distance of non-bond interaction was set at 10 Å, and the particle mesh Ewald (PME) algorithm was used to calculate the long-range electrostatic interaction [31,32]. The SHAKE algorithm constrained the hydrogen bond lengths, and the Langevin algorithm with a collision frequency γ of 2 ps^−1^ was selected [33,34]. Relevant trajectories were recorded every 10 ps with the system pressure at 1 atm and the integral step at 2 fs. According to the MM/GBSA method, the binding free energy of the PZ–FA complex could be calculated [35].

### 2.6. Iron Release Measurements from DSF in the Presence of Ferulic Acid and Beer

Measurements of iron release were performed as previously described with a few modifications [7]. The identification of iron release in the solvents was achieved through the absorption of [Fe (Ferrozine)_3_]^2+^ at 562 nm (ε_562_ = 27.9 mM^−1^cm^−1^) for 60 min using a UV-absorption spectroscopic measurement. The iron release system (0.5 mL) induced by ferulic acid includes a sodium citrate buffer (50.0 mM pH 4.2), DSF (0.1 μM), ferrozine (0.5 mM), and ferulic acid at a constant concentration of 320 μM with a different molar ratio than PZ. The iron release system (0.5 mL) induced by beer contains beer, DSF (0.1 μM), and ferrozine (0.5 mM) with a different molar ratio than PZ. To exclude the influence of PZ and as a buffer to iron measurement, the FeSO_4_ standard curve was obtained in the absence of DSF, and the groups contained control checks, buffers without PZ, and buffers with 14.5 μM of PZ. The control check contained 10–50 μM of FeSO_4_ and 0.5 mM of ferrozine, while other reaction systems contained 10~50 μM of FeSO_4_, 320 μM of FA, 50 mM of citrate, and 0.5 mM of ferrozine in the presence and absence of PZ. After the solution was prepared and developed color for 30 min, the absorbance value was determined at a wavelength of 562 nm.

### 2.7. Statistical Analysis

All experiments were conducted in triplicate. All data were presented as mean ± standard deviation (SD).

## 3. Results and Discussion

### 3.1. Purification and Characterization of PZ

**Figure 1:**
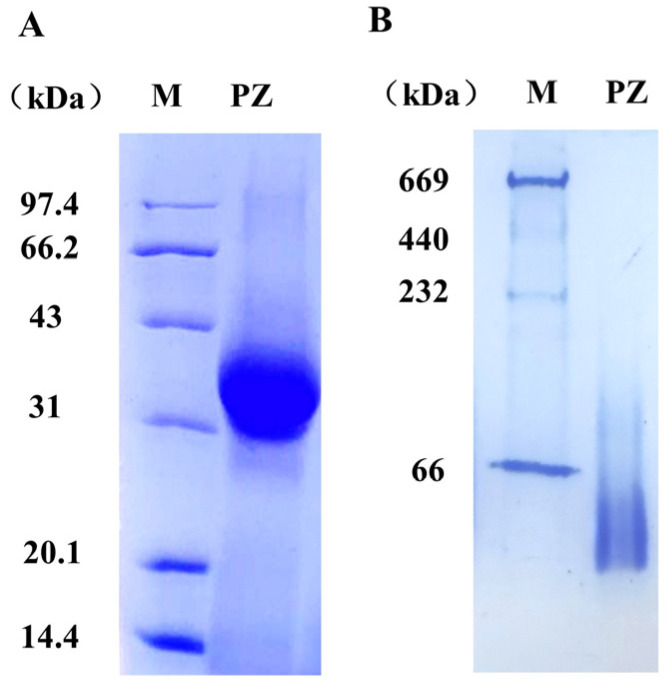
SDS-PAGE (**A**) analyses and native PAGE (**B**) analyses of PZ.

SDS-PAGE and native PAGE were used to separate and identify the purified PZ and DSF. As shown in Figure 1A,B, the PZ distribution was a single band on the SDS-PAGE and native-PAGE gel, and was estimated to be about 40 kDa, which is in accordance with the previous study [26]. The above findings revealed that the PZ was effectively purified as homogeneously pure and could be used for further experiment.

### 3.2. Interactions between PZ and FA

#### 3.2.1. The Stoichiometry and Binding Constant of PZ Binding with FA

**Figure 2:**
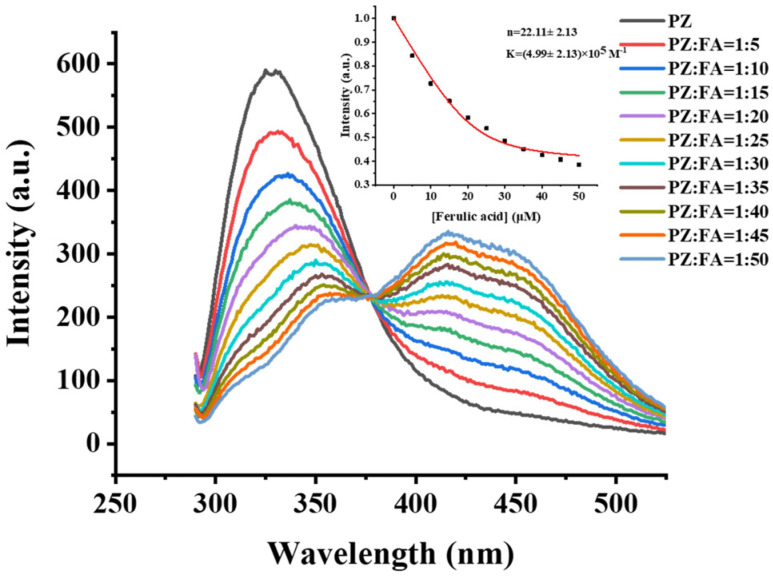
Fluorescence-quenching of PZ (1 μM) in the presence of FA with various molar ratios. The inset is the fit of Equation (1) to these data with n = 22.11 ± 2.13, K = 4.99 ± 2.13 × 10^5^ M^−1^. Conditions: λ_Ex_ = 280 nm, both slits for excitation and emission are 10 nm, 1.0 μM PZ, 5.0–50.0 μM FA, 50.0 mM sodium citrate buffer, pH 4.2, and 25 °C.

Fluorescence-quenching measurements have been widely used to investigate the binding interactions between proteins and small molecules [36]. Tryptophan (Trp) residues are considered the primary intrinsic fluorescence of proteins due to their sensitivity to the microenvironment surrounding fluorophore residues. There are three Trp residues in each PZ molecule, including Trp-150, Trp-186, and Trp-257. To determine the stoichiometry and binding constant, the PZ was titrated with FA gradually. In Figure 2, the emission fluorescence intensity decreased with an increasing concentration of FA, which indicated that there were interactions between PZ and FA. Meanwhile, a red shift was observed, and the maximum red shift reached 29 nm (from 327 to 356 nm), suggesting that the microenvironment around Trp residues changed after binding with ferulic acid, and Trp residues had been brought to a more hydrophilic environment [37].

The binding stoichiometry was established by fitting the fluorescence data to Equation (1):

(1) $$I=I_{0}-\frac{I_{0}-I_{\infty }}{2n{\left[P\right]}_{0}}\times \left[1/K+{\left[\mathrm{FA}\right]}_{0}+n{\left[P\right]}_{0}-\sqrt{{\left(1/K+{\left[\mathrm{FA}\right]}_{0}+n{\left[P\right]}_{0}\right)}^{2}-4n{\left[\mathrm{FA}\right]}_{0}{\left[P\right]}_{0}}\right]$$

In this equation, K is the binding constant, and I and I_0_ stand for the relative fluorescence intensities of PZ with and without FA at fully saturated sites, respectively. Here, [P]_0_ and [FA]_0_ represent concentrations of PZ and FA. From this equation, the value of K was calculated as (4.99 ± 2.13) × 10^5^ M^−1^, while one PZ molecule can bind 22.11 ± 2.13 of FA molecules (Figure 2, inset). The binding stoichiometry of PZ with FA is much higher than with curcumin, which may be associated with the different structures of FA and curcumin [25]. Furthermore, the binding constant of PZ–FA was in the order of 10^5^, which is significantly higher than PZ–xanthohumol (7.50 × 10^4^ M^−1^), human serum albumin–FA (2.23 × 10^4^ M^−1^) and alpha-2-macroglobulin–FA (5.83 × 10^4^ M^−1^), suggesting that the interaction between PZ and FA has a strong binding affinity [24,38,39].

#### 3.2.2. Analysis of the Binding Sites of Ferulic Acid on PZ

**Figure 3:**
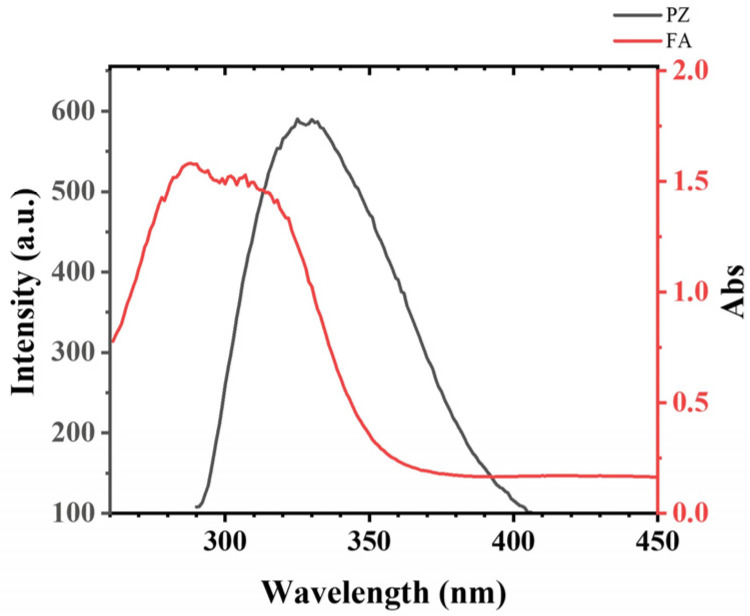
The overlap of the fluorescence emission spectrum of PZ with the absorption spectrum of FA. Conditions: λ_Ex_ = 280 nm, 1.0 μM PZ, 100.0 μM FA, 50.0 mM sodium citrate buffer, pH 4.2, and 25 °C.

According to Förster’s theory of non-radiation energy transfer, fluorescence resonance energy transfer (FRET) is considered a spectroscopic ruler for measuring the distance between the donor and acceptor [40]. FRET could happen as a result of the overlap between the fluorescence emission spectra of the donor and the UV–vis absorption spectra of the acceptor, and the distance is 2–8 nm [41]. As expected, there is good overlapping between the fluorescence emission spectrum of PZ and the absorption spectra of FA (Figure 3), indicating the occurrence of fluorescence resonance energy transfer (FRET) between PZ and FA. The distance between the donor (PZ) and acceptor (FA) can be determined as Equation (2) [42].

(2) $$E=1-\frac{F}{{\;F}_{0}}=\frac{R_{0}{}^{6}}{R_{0}{}^{6}+r^{6}}$$

where E is the energy transfer efficiency, R_0_ is the Förster distance at which half the energy is transferred, and r is the actual distance between donor and acceptor. F and F_0_ are the fluorescence intensities of PZ in the presence and absence of FA, respectively.

(3) $$R_{0}{}^{6}=8.79\times 10^{25}K^{2}Øn^{-4}J$$

Here, K^2^ is a factor of the transition dipoles describing the relative orientations of the donor and acceptor (a value of 2/3 for random orientation); Φ is the fluorescence quantum yield of the donor without acceptor (a value of 0.118 for tryptophan residues); n is the refractive index of the medium (the average value of water and organic solute is 1.366). J is the integral of overlapping areas between the fluorescence emission spectrum of the donor and the UV absorption spectrum of the acceptor, which can be approximated by:

(4) $$J=\frac{\int_{0}^{\infty }F\left(\lambda \right)\varepsilon \left(\lambda \right)\lambda^{4}d\lambda }{\int_{0}^{\infty }F\left(\lambda \right)d\lambda }$$

where F(*λ*) is the donor fluorescence intensity at wavelength *λ*, and *ε* is the molar absorption coefficient of the receptor at the wavelength of *λ*.

After calculation, J can be integrated according to Equation (4) (J = 8.74 × 10^−15^ cm^3^/M). R_0_ can be calculated using Equation (3) as 3.53 nm, and E can be obtained from Equation (2) as 69%. Finally, r was calculated to be 3.09 nm, which is less than 7 nm and 0.5 R_0_ < r < 2.0 R_0_. The result indicated that the binding distance between the Trp of PZ and FA is 3.09 nm.

#### 3.2.3. Effects of FA Binding on PZ Structure

**Figure 4:**
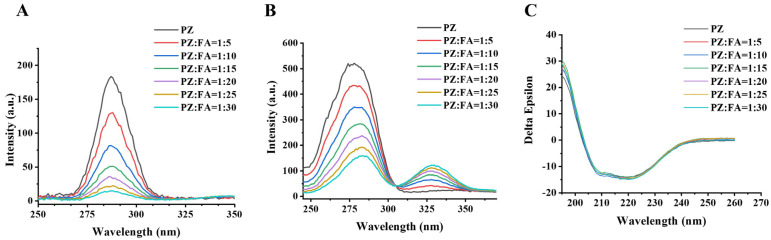
Synchronous fluorescence spectra of PZ (4 M) in the presence of FA with various molar ratios. (**A**) Δ*λ* = 15 nm; (**B**) Δ*λ* = 60 nm. Conditions: *λ*_Ex_ = 200 nm, slits for excitation and emission of 10 and 5 nm, respectively, 4 μM PZ, 20–120 μM FA, 50.0 mM sodium citrate buffer, pH 4.2, and 25 °C; (**C**) CD spectra of PZ in the presence of FA with various molar ratios. Conditions: the molar ratios of PZ (0.1 mg/mL, in 25.0 mM PB buffer, and pH 4.2) to FA varied from 1:5 to 1:30.

Despite the effect of FA binding on the tertiary structure of PZ, synchronous fluorescence spectra could provide the changes of the tertiary structure on Tyr and Trp residues when the Δ*λ* value is set at 15 nm or 60 nm, respectively [43]. As shown in Figure 4A,B, the synchronous fluorescence intensity of Tyr (Δ*λ* = 15 nm) and Trp (Δ*λ* = 60 nm) decreased gradually with the addition of FA, indicating that the conformation of PZ was changed, and both Tyr and Trp residues were involved in the quenching of the intrinsic fluorescence within PZ. Compared to Tyr, the decrease in synchronous fluorescence intensity on Trp is more significant, suggesting that the binding site of the PZ–FA interaction was close to Trp residues. This result is in accordance with that calculated from FRET. Furthermore, a red shift of the Trp residue fluorescence spectra (from 277 to 283) was observed, which reveals that the polarity around the tryptophan residues was more hydrophilic.

In addition, the effect of FA on the secondary structure has been identified. Figure 4C shows the far-UV CD spectra of PZ in the absence and presence of FA, and there were two negative bands around 208 and 222 nm as well as a strong positive band at 200 nm, which is the characteristic of α-helix and β-sheet structures [44]. However, after the addition of FA, the negative ellipticity, the shape of the peaks, and the position of the peak maximum did not change, which illustrated that the binding of FA did not change the secondary structure of PZ.

### 3.3. MD Analysis

**Figure 5:**
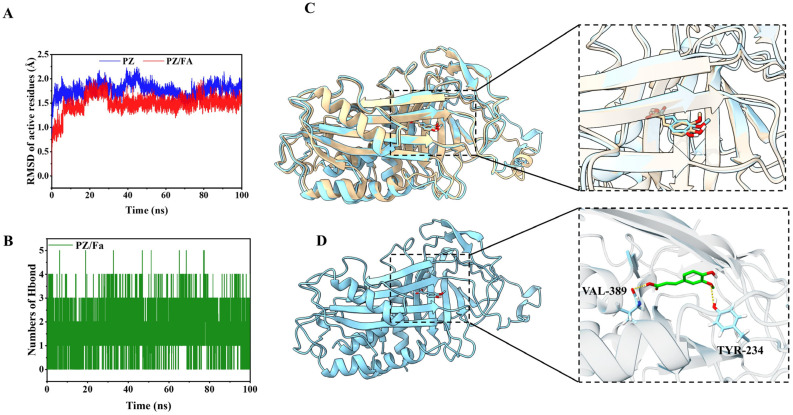
(**A**) Root-mean-square deviation (RMSD) of active sites over time during MD simulations; (**B**) changes in the number of hydrogen bonds formed by PZ–FA during MD; (**C**) comparison of the conformation of PZ–FA complex before and after MD simulations—the yellow represents the conformation before simulation, and the blue represents the last frame of MD; (**D**) the binding situation of PZ-FA. The yellow dotted line shows the hydrogen bonds.

MD analysis was performed to obtain the potential PZ–FA binding sites and forces related to their interactions, which was on the basis of lowest energy conformation from molecular docking. The root-mean-square deviation (RMSD) can reflect the motion process of the protein–small molecule complex, and the fluctuation means the motion is violent; otherwise, the motion is stable. The RMSD of the active sites of PZ [45] was monitored, and it is worth noting that the active sites tended to be stabilized with FA binding, which indicated that the FA molecule could stabilize the site, and there is a good binding effect between PZ and FA (Figure 5A).

The binding free energy predicted using the MM-GBSA method was (−25.20 ± 2.18) kcal/mol (Table S1), which implied that FA could form an affinity with PZ. Additionally, the leading contribution of PZ–FA binding is van der Waals energy (−28.88 ± 2.04 kcal/mol), followed by electrostatic energy (−21.30 ± 2.77 kcal/mol), and then the non-polar contribution to solvation (−4.61 ± 0.05 kcal/mol). This result correlated well with the ovalbumin–FA interactions, and its binding free energy reached −26.78  kcal/mol [46]. For further analysis of binding energy, we explored the contribution of amino acids to binding. As shown in Figure S1, residues Val 337, Phe 388, Glu 338, Val 389, Phe 376, Ala 39, Ile 378, Phe 182, Val 339, and Met 245 are the ten amino acids that contribute the most to the binding of PZ–FA, particularly Val 337 and Phe 388.

Herein, we compared the changes in conformation of the PZ–FA complex before and after MD in Figure 5C; the loop around the active site of PZ changes into a more stable β-sheet, which is consistent with the above RMSD results. This may be brought about by the binding with FA. After MD, FA was embedded in the internal hydrophobic active pocket of PZ (Figure S2), and the binding situation is shown in Figure 5D; there were two hydrogen bonds between FA and Val 389 and Tyr 234, respectively. It is suggested that these two residues played significant roles in the formation of hydrogen bonds. The numbers of hydrogen bonds within 100 ns during MD were recorded in Figure 5C, illustrating that there were 1–3 hydrogen bonds maintained between FA and PZ during the MD process. The results were in accordance with the form research, which had shown 2–3 hydrogen bonds between PZ and curcumin, and 0–4 hydrogen bonds between ovalbumin and FA [25,46]. As one of the main non-covalent interactions, hydrogen bonds play a crucial role in non-covalent interactions of the PZ–FA complex.

### 3.4. Inhibition of Iron Release through PZ–FA Interactions

The results above studied the interaction between PZ and FA. To verify the assumption that their interaction would inhibit iron release from ferritin, naturally-occurring ferritin from donkey spleen was isolated and purified. DSF was distributed around 19.5 kDa and 21 kDa, as shown in Figure S3A, which indicated that DSF was composed of two subunits. Native PAGE of DSF in Figure S3B illustrated that the molecular weight of DSF is ~440 kDa. Subsequently, we explored the iron release induced by FA and beer solutions in the presence and absence of PZ. In addition, it is proved that the iron release measurement by ferrozine was not affected by PZ and buffers. In Figure S4, there was no significant difference among the three groups when the concentration of FeSO_4_ was 10, 20, 30, and 50 μM. Therefore, the effect of PZ and buffers on the iron measurement is negligible, and the iron release measurement method used in this work is feasible. According to the fluorescence-quenching measurements that one PZ could bind 22 FA molecules, the molar ratios of PZ and FA were chosen at 1:11, 1:22, 1:44, and 1:66. To evaluate the initial rate of iron release, the kinetic curve was fitted using a third-order equation based on the previous report:

Y = A_0_ + A_1_t + A_2_t^2^ + A_3_t^3^ and dY/dt = A_1_+ 2A_2_t + 3A_3_t^2^ (at t = 0, (dY/dt)_0_ = v_0_). (5)

**Figure 6:**
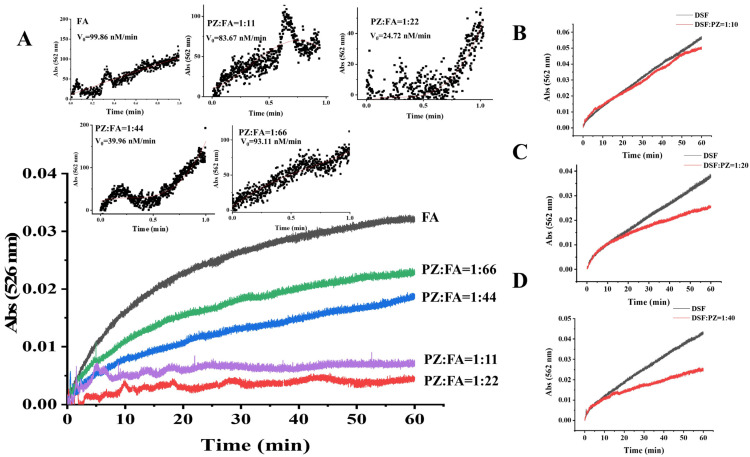
(**A**) Iron release curve of DSF induced by FA. Iron release from DSF was measured at 562 nm for 60 min due to the chelation of Fe^2+^ by ferrozine. Conditions: 320 μM FA with varieties of PZ (from 1:11 to 1:66, PZ:FA molar ratio), 0.1 μM DSF, and 0.5 mM ferrozine. Inset is the fitted curve of initial release rate. Iron release of DSF in beer when molar ratio of DSF:PZ is 1:10 (**B**), 1:20 (**C**), and 1:40 (**D**). Conditions: 0.1 M DSF and 0.5 mM ferrozine.

In this equation, t stands for time, and Y is the concentration of [Fe(ferrozine)_3_]^2+^ at t minutes [47]. As illustrated in Figure 6A, when PZ: FA was at 1:22, 1:44, and 1:66, the initial release rate of iron decreased to 24.72, 39.96, and 93.11 nM/min, respectively, compared to without PZ addition (99.86 nM/min). At 60 min, they released 0.16 μM, 0.67 μM, and 0.82 μM iron, respectively, which is much lower than FA without PZ (1.16 μM). In addition, the amount of released iron induced by FA with PZ:FA at 1:22, 1:44, and 1:66 after 60 min of incubation decreased by 86.20%, 42.24%, and 29.31%, respectively. When PZ: FA was at 1:22, the content of iron release was the least, suggesting that the PZ–FA interaction effectively inhibited the iron release. In our study, the iron release from DSF was not inhibited efficiently at the ratio of 1:11 (PZ and FA), which may be due to the fact that PZ molecules may assemble together when concentration increases, and thus affected the interaction between PZ and ferulic acid.

The beer contained plenty of polyphenols such as gallic acid, salicylic acid, chlorogenic acid, vanillic acid, and ferulic acid [48]. Thus, we further tested the inhibition effect of PZ in beer systems. As shown in Figure 6B–D, the amount of released iron reached 1.78 μM, 0.92 μM, and 0.88 μM with DSF:PZ at 1:10, 1:20, and 1:40, and the iron release decreased by 11.52%, 33.42%, and 40.5%, respectively. Inspired by this, the addition of stable beer-occurring PZ would interact with the polyphenols, by which the iron release could be prevented. The inhibition of iron release is meaningful for the application of ferritin. It will protect sensitive components in food substances, such as lipids, from oxidation and degradation by Fe^2+^, which contributes to an iron smell and discoloration.

## 4. Conclusions

Protein–small molecule interactions commonly occur in foodstuffs, which could lead to either benefits or harm to the quality of food. Ferritin is vulnerable to iron release by multiple reductants in food, which makes it challenging for further applications. In this study, we explored a novel method based on protein–polyphenol interactions to reduce iron release. The interactions between the malt-derived PZ and FA were evaluated using muti-spectroscopy methods, and we found that one PZ molecule can bind with 22.11 ± 2.13 of FA with the binding constant at (4.99 ± 2.13) × 10^5^ M^−1^, while the tryptophan residues of PZ were made more hydrophilic without changing the secondary structure. FRET indicated that the binding distance between PZ and FA is 3.09 nm. In addition, MD analysis showed that FA could enter the internal hydrophobic pocket of PZ and form hydrogen bonds with Val-389 and Tyr-234. As expected, the iron release from DSF in beer showed a decrease of up to 40.5% in the presence of PZ. In FA solutions, the iron release was reduced by 86.20% when PZ:FA was 1:22 (molar ratio) compared with that without PZ addition. In conclusion, the inhibition of iron release from DSF is effective based on the PZ–FA interaction, and it will provide a new approach for the prevention of iron release from ferritin in foodstuffs.

## Data Availability

The data are included within the article.

## Supplementary Materials

The following supporting information can be downloaded at: https://www.mdpi.com/article/10.3390/foods12020234/s1, Figure S1: The amino acids that contribute most to binding energy; Figure S2: The model of PZ–FA complex after MD. Dark cyan stands for the hydrophilic area and yellow stands for hydrophobic area; Figure S3: SDS-PAGE (A) analyses and native PAGE (B) analyses of DSF; Figure S4: The comparison of standard curve in the presence and absence of PZ. Control check: 10–50 μM FeSO_4_ and 0.5 mM ferrozine; Buffers without PZ: 10–50 μM FeSO_4_, 320 μM FA, 50 mM citrate and 0.5 mM ferrozine; Buffers with PZ: 10–50 μM FeSO_4_, 320 μM FA, 50 mM citrate, 0.5 mM ferrozine and 14.5 μM PZ. The iron was measured at a wavelength of 562 nm; Table S1: Binding free energies and energy components predicted by MM/GBSA (kcal/mol).
