# Supplementary material for: Inhibition of Iron Release from Donkey Spleen Ferritin through Malt-Derived Protein Z–Ferulic Acid Interactions
Source: Foods. 2023 Jan 4;12(2):234. doi: 10.3390/foods12020234 (PMC9857996; doi:10.3390/foods12020234)
Supplement: Supplementary file 1 [file foods-12-00234-s001.zip › foods-2148467-supplementary.pdf]

Inhibition of Iron Release from Donkey Spleen Ferritin through Malt-Derived Protein Z–Ferulic Acid Interactions

Mingyang Sun, Hanhan Liu, Chen Xu, Zhenghui Jiang and Chenyan Lv *

College of Food Science & Nutritional Engineering, China Agricultural University, Beijing Key Laboratory of Functional Food from Plant Resources, Beijing 100083, China

Corresponding Author:

Chenyan Lv, PhD

No. 17 Qing Hua East Road, Haidian District, Beijing, China

E-mail: 2019023@cau.edu.cn

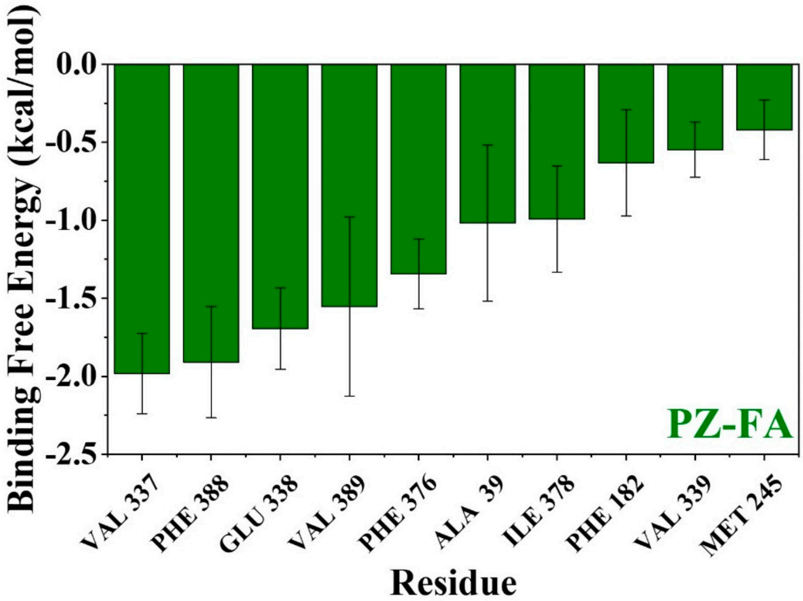

Figure S1. The amino acids that contribute most to binding energy.

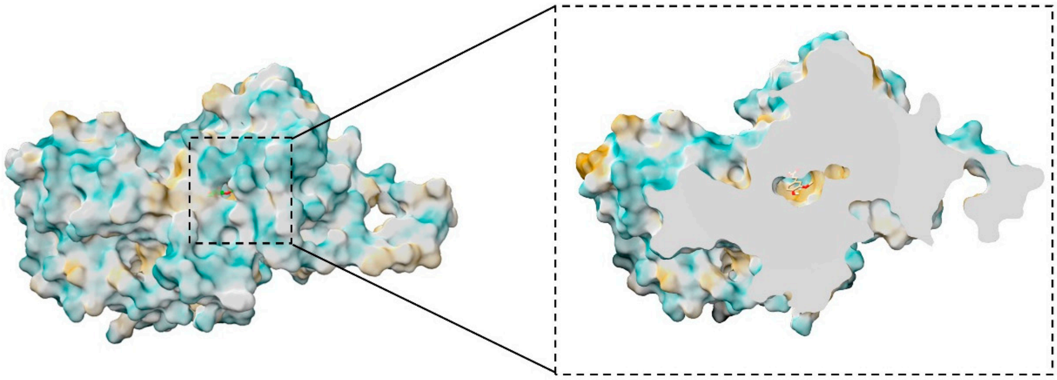

Figure S2. The model of PZ-FA complex after MD. Dark cyan stands for the hydrophilic area and yellow stands for hydrophobic area.

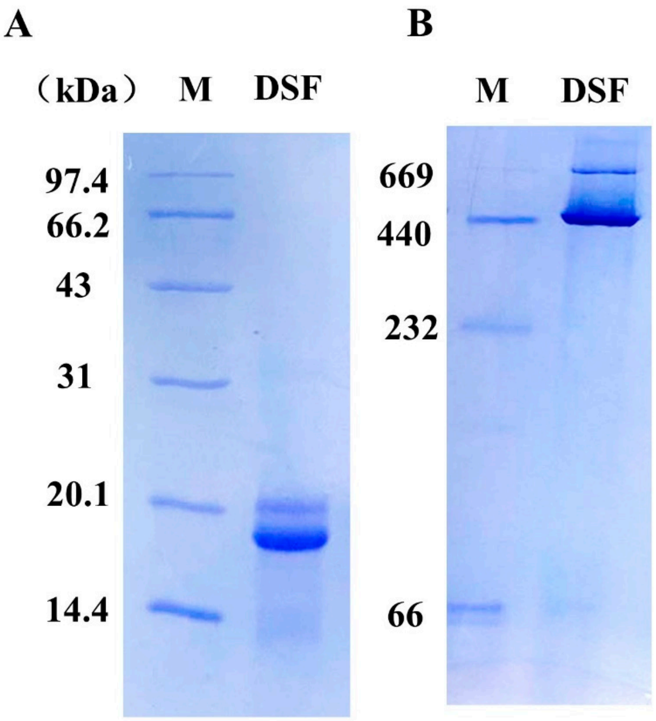

Figure S3. SDS-PAGE (A) analyses and native-PAGE (B) analyses of DSF.

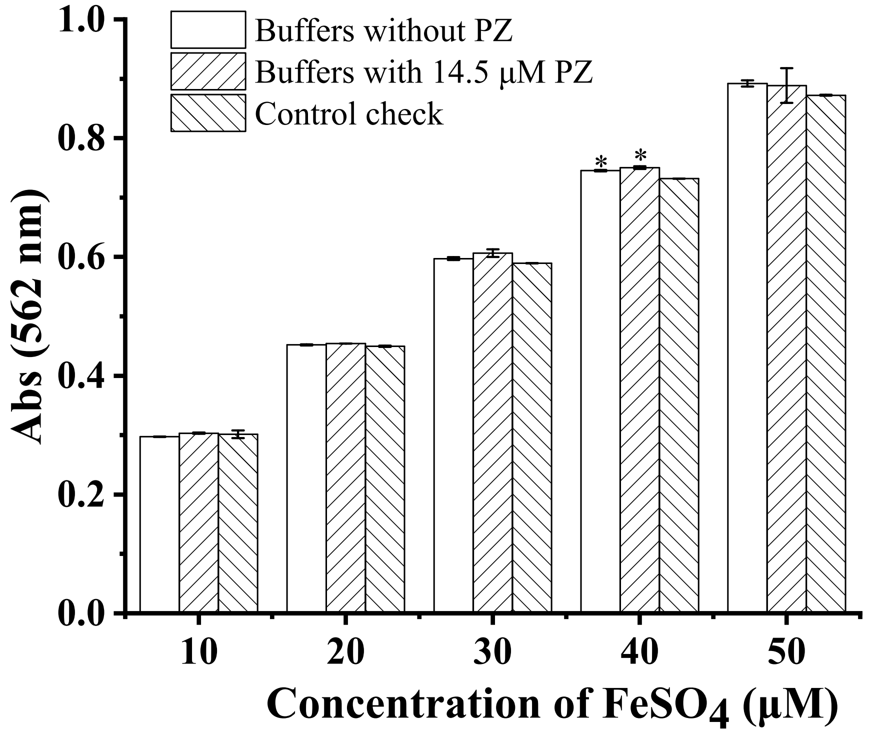

Figure S4. The comparison of standard curve in the presence and absence of PZ. Control check: 10-50 μM $FeSO_4$ and 0.5 mM ferrozine; Buffers without PZ: 10-50 μM $FeSO_4$, 320 μM FA, 50 mM citrate and 0.5 mM ferrozine; Buffers with PZ: 10-50 μM $FeSO_4$, 320 μM FA, 50 mM citrate, 0.5 mM ferrozine and 14.5 μM PZ. The iron was measured at a wavelength of 562 nm.

Table S1. Binding free energies and energy components predicted by MM/GBSA (kcal/mol).

**System name**	PZ-fa
**ΔE_{vdw}**	-28.88 ±2.04
**ΔE_{elec}**	-21.30±2.77
**ΔG_{GB}**	29.59±0.89
**ΔG_{SA}**	-4.61±0.05
**ΔG_{bind}**	-25.20 ±2.18

ΔE_{vdW}: van der Waals energy.

ΔE_{elec}: electrostatic energy.

ΔG_{GB}: electrostatic contribution to solvation.

ΔG_{SA}: non-polar contribution to solvation.

ΔG_{bind}: binding free energy.
